# Field comparison of OraQuick® ADVANCE Rapid HIV-1/2 antibody test and two blood-based rapid HIV antibody tests in Zambia

**DOI:** 10.1186/1471-2334-12-183

**Published:** 2012-08-08

**Authors:** Dalila Zachary, Lawrence Mwenge, Monde Muyoyeta, Kwame Shanaube, Albertus Schaap, Virginia Bond, Barry Kosloff, Petra de Haas, Helen Ayles

**Affiliations:** 1 ZAMBART Project, University of Zambia, Nationalist Road, Ridgeway, Lusaka, Zambia; 2 Miriam Hospital, Warren Alpert Brown University School of Medicine, Providence, RI, USA; 3 Department of Clinical Research, London School of Hygiene & Tropical Medicine, London, UK; 4 Department of Global Health and Development, London School of Hygiene & Tropical Medicine, London, UK

**Keywords:** HIV, Zambia, OraQuick®, Cost

## Abstract

**Background:**

Zambia’s national HIV testing algorithm specifies use of two rapid blood based antibody assays, Determine®HIV-1/2 (Inverness Medical) and if positive then Uni-Gold^TM^ Recombigen HIV-1/2 (Trinity Biotech). Little is known about the performance of oral fluid based HIV testing in Zambia. The aims of this study are two-fold: 1) to compare the diagnostic accuracy (sensitivity and specificity) under field conditions of the OraQuick® ADVANCE® Rapid HIV-1/2 (OraSure Technologies, Inc.) to two blood-based rapid antibody tests currently in use in the Zambia National Algorithm, and 2) to perform a cost analysis of large-scale field testing employing the OraQuick®.

**Methods:**

This was a operational retrospective research of HIV testing and questionnaire data collected in 2010 as part of the ZAMSTAR (Zambia South Africa TB and AIDS reduction) study. Randomly sampled individuals in twelve communities were tested consecutively with OraQuick® test using oral fluid versus two blood-based rapid HIV tests, Determine® and Uni-Gold^TM^. A cost analysis of four algorithms from health systems perspective were performed: 1) Determine® and if positive, then Uni-Gold^TM^ (Determine®/Uni-Gold^TM^); based on current algorithm, 2) Determine® and if positive, then OraQuick® (Determine®/OraQuick®), 3) OraQuick® and if positive, then Determine® (OraQuick®/Determine®), 4) OraQuick® and if positive, then Uni-Gold^TM^ (OraQuick®/Uni-Gold^TM^). This information was then used to construct a model using a hypothetical population of 5,000 persons with varying prevalence of HIV infection from 1–30%.

**Results:**

4,458 participants received both a Determine® and OraQuick® test. The sensitivity and specificity of the OraQuick® test were 98.7 (95%CI, 97.5–99.4) and 99.8 (95%CI, 99.6–99.9), respectively when compared to HIV positive serostatus. The average unit costs per algorithm were US$3.76, US$4.03, US$7.35, and US$7.67 for Determine®/Uni-Gold^TM^, Determine®/OraQuick®, OraQuick®/Determine®, and OraQuick®/Uni-Gold^TM^, respectively, for an HIV prevalence of 15%.

**Conclusions:**

An alternative HIV testing algorithm could include OraQuick® test which had a high sensitivity and specificity. The current Determine®/Uni-Gold^TM^ testing algorithm is the least expensive when compared to Determine®/OraQuick®, OraQuick®/Determine®, and OraQuick®/Uni-Gold^TM^ in the Zambian setting. From our field experience, oral fluid based testing offers many advantages over blood-based testing, especially with self testing on the horizon.

## Background

The introduction of rapid HIV antibody tests has revolutionized HIV diagnosis by facilitating the testing of millions of people worldwide. The availability of affordable, point-of-service HIV testing is especially important in low-income, high-HIV-burden countries which lack the financial and technological resources to perform more sophisticated laboratory-based assays. For these reasons, blood based rapid HIV tests have become the standard of care and the basis for the national HIV testing algorithm in many developing countries, including Zambia [1-3].

Zambia’s HIV prevalence of 13.5% [4] makes HIV testing a national health priority and the national HIV testing algorithm specifies sequential blood-based rapid antibody tests: first Determine®HIV-1/2 Antibody (Inverness Medical) is used, which if reactive is followed by a different antibody test, Uni-Gold^TM^ Recombigen HIV-1/2 Antibody (Trinity Biotech). If the screening and confirmatory tests yield discordant results, then a third blood-based rapid antibody test, Bioline® HIV-1/2 test (Standard Diagnostic), is performed or blood is sent for enzyme linked immunosorbent assay (ELISA) testing [1].

Despite the benefits of HIV testing, a majority of patients living in developing countries are unaware of their status. Nine countries, seven of which were in sub-Saharan Africa (Democratic Republic of Congo, Kenya, Liberia, South Africa, Swaziland, Tanzania, Zambia), conducted population-based surveys during 2007 and 2008 and reported HIV testing rates. Collectively, these seven countries account for 32% of the people living with HIV globally and 45% of those in sub- Saharan Africa. Among the seven countries in sub-Saharan Africa, the median HIV testing rates were 30% among women and 17% among men. In Zambia, 64.7% of women and 79.2% of men reported never having an HIV test [5-9]. In Zimbabwe, inconvenience of testing location and testing hours were reported as the main reasons for individuals not accessing voluntary counseling and testing (VCT) services [10]. These data underscore the challenges of implementing HIV testing and prevention that are broadly available and accessible in developing countries and furthermore suggest the need for innovations in this field.

As useful as blood-based rapid HIV tests have proven to be, it may be possible to further expand and improve HIV testing services by employing rapid tests using oral fluid. Both oral fluid based and blood based rapid HIV tests are performed manually and visually read; however, oral fluid-based HIV tests offer several advantages over blood based assays: oral fluid collection is less invasive as it does not require blood draw or finger stick, can be used to self test and is less hazardous because oral fluid has a lower transmission risk of HIV compared to blood [11-13]. Additionally, in consideration of oral fluid based testing as a practical alternative, there are potential cost limitations. There are few studies that address the cost of oral based testing in the field; however, the slow uptake of oral HIV testing by resource constrained countries may be due to the cost per test, which is higher than most blood based tests [14]. Moreover, oral fluid based HIV testing performance and sensitivity in the developing countries [15-20] and cross reactivity with other infections like dengue fever [21] are still being published.

In this study, we performed a field comparison of OraQuick® ADVANCE Rapid HIV-1/2 Antibody test (OraSure Technologies, Inc.) to two blood-based rapid antibody tests currently in use in the Zambian National Algorithm for HIV testing. The objectives of this study were two-fold: 1) to compare the diagnostic accuracy (sensitivity and specificity) under field conditions of the OraQuick® using oral fluid to two blood-based rapid antibody tests currently in use in the Zambia National Algorithm, and 2) to perform a cost analysis of large-scale field testing employing the OraQuick® test.

## Methods

During 2009 and 2010, the Zambia South Africa TB and AIDS reduction study (ZAMSTAR) conducted a TB and HIV prevalence survey among 59,705 randomly sampled adult residents of sixteen communities in Zambia [22]. The ZAMSTAR study was a community randomized trial with the main objective of reducing TB and HIV prevalence by implementing two community level interventions. Within this larger study, we nested a field comparison trial of OraQuick® test using oral fluid versus two blood-based rapid HIV tests, Determine® and Uni-Gold^TM^. Approximately 4,900 OraQuick® tests were available for use in the field.

### Study setting

**Figure 1:**
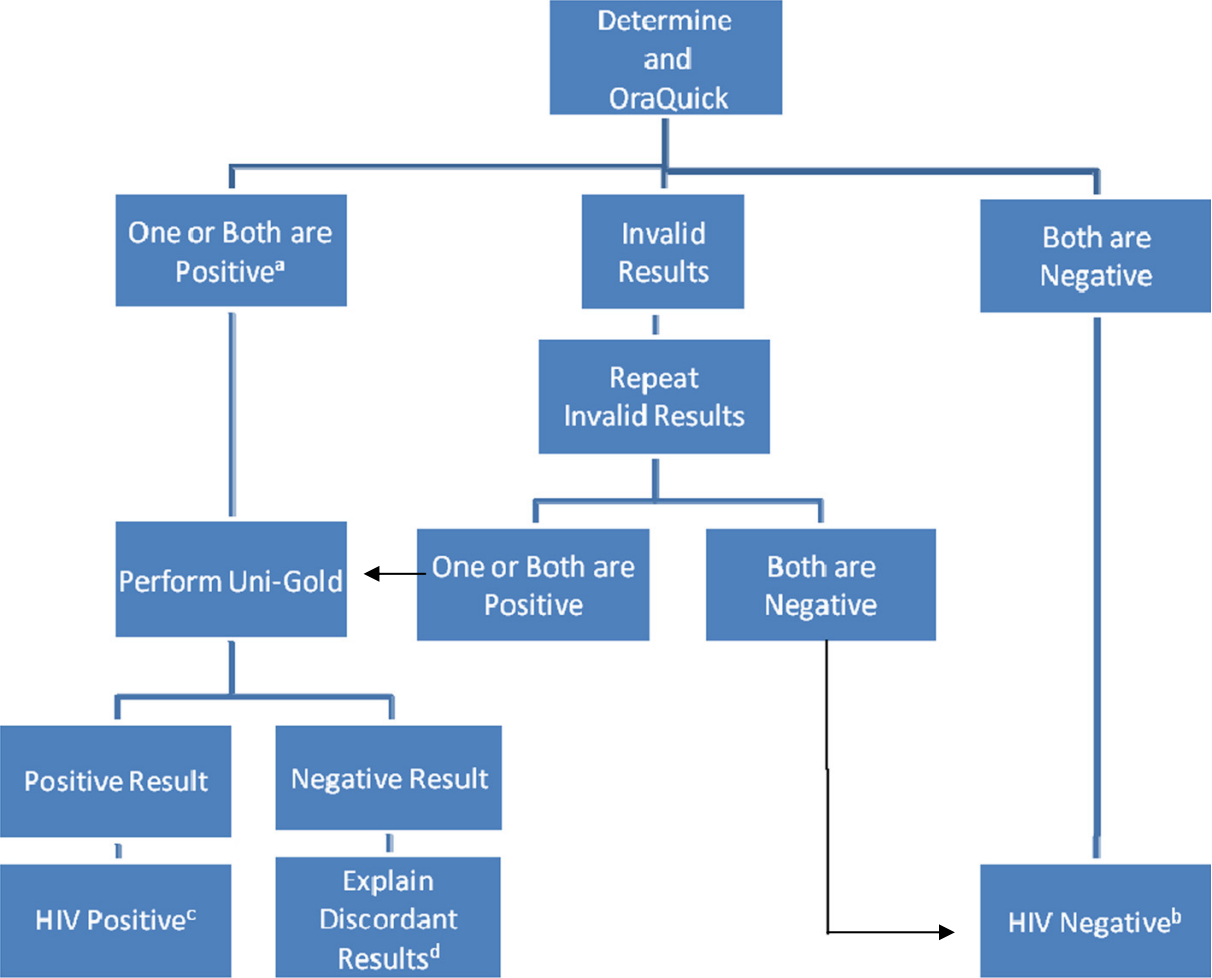
**HIV testing algorithm.** A If patient self reported Positive HIV status prior to testing then Uni-Gold^TM^ testing was not performed and patient was referred to local ART clinic. B Patient told about the “window period” and asked to repeat test in 3 months. C Patient was referred for care at local ART clinic. D Patient was referred for further testing via routine VCT centers.

Of the sixteen ZAMSTAR study communities, twelve were conveniently sampled to take part in this field comparison study. Randomly sampled individuals in twelve communities in Zambia were tested consecutively from April 29, 2010 to August 13, 2010 in their homes for HIV using both the oral fluid and blood-based rapid assays (Figure 1). All steps in the HIV testing process (pre-test counseling, sample collection, performance of assays, interpretation of results, presentation of results and post-test counseling) were performed in the participants’ homes by qualified counselors. The twelve selected sites were mostly urban communities and one rural community.

### Study enrolment/selection criteria

Participation in this field comparison study was open to all individuals who met inclusion criteria for the tuberculosis prevalence survey. The inclusion criteria for the tuberculosis prevalence survey were: any person living in one of the 12 demarcated communities where the study was taking place, at least 18 years of age, able to give informed consent and able to provide a sputum sample.

### Performing the test

HIV testing was offered by trained HIV counselors. Specific training on how to use OraQuick® test kit was offered to 4 team leaders by certified trainers from the local OraQuick® manufacturing and distributing company. In the field, 48 counselors were trained on how to use the OraQuick® test kit by the team leaders.

A questionnaire was administered and then participants were offered OraQuick® test in parallel to Determine® test with an explanation that we were performing a field trial of an HIV test that is not ordinarily used in our setting (Figure 1). We explained that the information collected would be useful in informing policy makers on the performance of the oral based rapid HIV test and its potential use in the future.

All testing was performed according to the manufacturers’ instructions [23-25]. The OraQuick® package instructions direct the person to place the flat pad above the teeth against the outer gum. The person gently swabs around the outer gums, both upper and lower, one time around, using the flat pad. The person should not swab the roof of the mouth, the inside of the cheek or tongue. The flat pad is then inserted into the developer solution vial and results can be read between 20 and 40 minutes. Counselors were instructed during their training that they may need to wait longer than 40 minutes and that the band may be faint on the OraQuick® test if the patient was HIV infected and taking antiretroviral therapy. Participants who reported HIV infection prior to testing were not tested using Uni-Gold^TM^ if the first test (Determine® or OraQuick®) was positive.

### Data collection and analysis

HIV test results were entered immediately into personal digital assistants (PDA) by the counselor who had performed the test. Participants with a positive Determine® and Unigold^TM^ test were given a referral slip to the local health facility to access care. Study data, including the questionnaire were conveyed to the main office in Lusaka, where they were downloaded and analyzed using Stata v. 11 (StataCorp, College Station, TX, USA). All tests which were recorded as being done within testing period of April 29, 2010 to August 13, 2010 were included in the analysis. Variables analyzed include baseline demographics and rapid test results. Measurement of each tests’ sensitivity and specificity were compared using *X*^2^ test and reported with 95% confidence intervals. Sensitivity of OraQuick® was measured compared to HIV positive serostatus. HIV positive serostatus was defined by either having both a positive Determine® and Uni-Gold^TM^ test result or a positive Determine® test result when the patient self reported a positive HIV serostatus prior to HIV testing.

We performed a cost analysis of four algorithms from health systems perspective [26]: 1) Determine® and if positive, then Uni-Gold^TM^ (Determine®/Uni-Gold^TM^); based on current algorithm, 2) Determine® and if positive, then OraQuick® (Determine®/OraQuick®), 3) OraQuick® and if positive, then Determine® (OraQuick®/Determine®), 4) OraQuick® and if positive, then Uni-Gold^TM^ (OraQuick®/Uni-Gold^TM^). Program costs were retrogressively collected including personnel for testing and counseling, HIV rapid test kits, medical and non-medical equipment and supplies, e.g. gloves, sharps container, lancets, and test kits. Assumptions were built into each respective algorithm regarding the infrastructure and overhead costs. First assumption was that these tests are done under field conditions, where counselors perform the test in the client’s home. Second assumption was that operation costs, for example utilities (electricity and water), space and maintenance, were the same across all tests. Third assumption was that the set-up costs were the same for all the tests, e.g., training of HIV counselors. Cost of an algorithm was estimated based on the cost of screening a sample on the first rapid HIV assay and confirmation of a reactive sample on the second rapid HIV assay. This information was then used to construct a model using a hypothetical population of 5,000 persons with varying prevalence of HIV infection from 1–30%. The published sensitivity and specificity values [23-25] for each test were used to calculate the number of expected positive tests for each prevalence category in the model and cost per algorithm was reported in terms of average cost per case.

### Ethics

This study received ethical approval from the University of Zambia biomedical research ethics committee.

## Results

**Figure 2:**
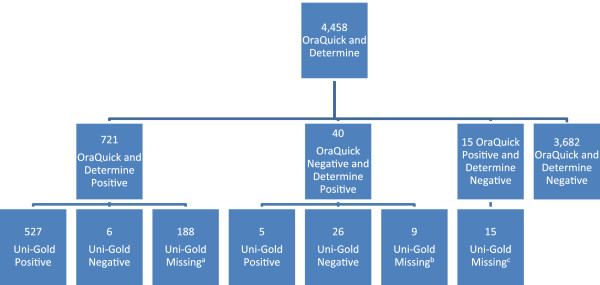
**OraQuick® and Determine® testing.** A 138 reported HIV positive status prior to testing. B 4 reported HIV positive status prior to testing. C 3 reported HIV positive status prior to testing.

4,458 participants received both a Determine® and OraQuick® test (Figure 2). 3,682 (82.6%) had both a negative OraQuick® and Determine® test result. 721 (16.2%) participants had both a positive Determine® and OraQuick® test result. The sensitivity and specificity of the OraQuick® test were 98.7 (95%CI, 97.5–99.4) and 99.8 (95%CI, 99.6–99.9), respectively when compared to HIV positive serostatus (Table 1).

**Table 1:** Sensitivity and specificity of OraQuick® test

**Compared to HIV infected**^**1**^**, N = 4,391**	
Sensitivity (95% CI)	98.7% (97.5%–99.4%)
Specificity (95% CI)	99.8% (99.6%–99.9%)
Positive Predictive Value (95%CI)	99.1% (98.1%–99.7%)
Negative Predictive Value (95%CI)	99.8% (99.5%–99.9%)

^1^HIV infected as defined by both a positive Determine® and Uni-Gold^TM^ test result and/or self reported positive status prior to testing with a positive Determine® or OraQuick® test result.

There were 55 discordant Determine® and OraQuick® test results. 40 (73%) were OraQuick® negative and Determine® positive and 15 (27%) were OraQuick® positive and Determine® negative. A Uni-Gold^TM^ test should have been performed when either the Determine® or OraQuick® tests result were positive, unless the participant reported a positive HIV status prior to testing; however, 31 (56%) Uni-Gold^TM^ test results were available for the 55 discordant test results. 7 (13%) reported a positive HIV status prior to testing. Of the 31 Uni-Gold^TM^ test results available for discordant Determine® and OraQuick® test results, Uni-Gold^TM^ had a higher agreement rate with OraQuick® test results compared to Determine® test results. 26 (84%) Uni-Gold^TM^ and OraQuick® negative test results agreed and 5 (16%) Uni-Gold^TM^ and Determine® positive test results agreed. Female gender was associated with discordant test results, OR = 2.5, 95%CI: 1.2–5.2. Age, community, HIV status, and antiretroviral use were not associated with discordant test results.

When the HIV prevalence is 15%, the average unit costs per algorithm were US$3.76, US$4.03, US$7.35, and US$7.67 for Determine®/Uni-Gold^TM^, Determine®/OraQuick®, OraQuick®/Determine®, and OraQuick®/Uni-Gold^TM^ algorithms, respectively. The cost analysis shows that Determine®/Uni-Gold^TM^ was the lowest cost option when compared to the other testing algorithms, irrespective of the HIV prevalence (Table 2).

**Table 2:** Average unit cost^*^of 6 algorithms at various HIV prevalence rates in a hypothetical population of 5,000 persons

**Algorithm**	**Average Cost (US$) at different HIV Prevalence**						
	**1%**	**5%**	**10%**	**15%**	**20%**	**25%**	**30%**
Determine®/Uni-Gold^TM^	3.28	3.42	3.59	3.76	3.92	4.09	4.26
Determine®/OraQuick®/	3.31	3.52	3.77	4.03	4.29	4.55	4.81
OraQuick®/Determine®	7.17	7.22	7.29	7.35	7.41	7.48	7.54
OraQuick®/Uni-Gold^TM^	7.20	7.33	7.50	7.67	7.83	8.00	8.17

^*^Unit cost represents the cost screening HIV in each algorithm.

## Discussion

OraQuick® ADVANCE Rapid HIV-1/2 Antibody test using oral fluid performed well under field conditions in Zambia with a sensitivity of 98.7 (95%CI, 97.5–99.4) and specificity of 99.8 (95%CI, 99.6–99.9). The high sensitivity and specificity rates in Zambia are comparable to Pascoe et al. study evaluating OraQuick® in the field and in rural Zimbabwe [18]. A meta-analysis by Pai et al., reported a sensitivity of 98.03%[27] for OraQuick® testing, which is also comparable to our findings.

Not only did OraQuick® perform well under field conditions in Zambia, it also had several notable advantages over blood based rapid HIV testing such as elimination of discomfort of a finger prick and reduction of blood exposure for the health care worker. Anecdotally, many non-clinical staff found the kits easy to use as well as to train others to use. The participants were curious about the new technique, but overall the oral fluid based HIV testing was widely accepted and did not seem to confuse participants about the transmission of HIV through oral secretions; however, a separate study would need to be performed to address these issues more clearly.

Proper storage of rapid HIV test kits is also essential to ensure optimal performance. This can be challenging in climates with extreme temperatures. The manufacturers’ recommended storage temperatures for the test kits discussed in this paper are similar: OraQuick® should be stored at 15°–27°C, Uni-Gold^TM^ should be stored at 2°–27°C, and Determine® should be stored at 2°–30°C [23-25]. While these wide temperature ranges simplify storage and transport at ambient temperatures in mild or moderate climates, they do not preclude the need for air-conditioned rooms, refrigerators and transport coolers in hot climates. Our review of the literature did not find any studies specifically examining the performance of OraQuick® when stored above 27°C.

Although there are several published studies validating the OraQuick® test in Africa, there are few studies that address the cost of oral based rapid HIV testing in the field. There is one study in Ethiopia which performed a direct cost comparison between Determine®, OraQuick®, Capillus® HIV-1/HIV-2 Rapid Test Kit (Trinity Biotech) and Uni-Gold^TM^. The authors of this study suggested implementing oral based tests in Ethiopia’s national algorithm. Determine®, Capillus®/OraQuick® (presence/absence of refrigeration) and Uni-Gold^TM^ were recommended as screening, confirmatory and tiebreaker tests, respectively [28]. Another study inTanzania formulated an alternative cost beneficial confirmatory HIV rapid testing algorithm[29]. They included rapid HIV tests according to WHO recommendation criteria of cost per test < US$2. Both the Ethiopian and Tanzanian studies did not clearly state the costing methods or show the cost findings. Our study found that Determine®/Uni-Gold^TM^ testing algorithm is the most attractive investment of the four algorithms examined, followed by Determine®/OraQuick® algorithm. However, the Determine®/OraQuick® algorithm is not ideal practically because it loses the main advantage of having OraQuick® as a screening test, which is that it can be used for self testing. In developed countries, like the United States, there are more studies looking at cost for oral based rapid HIV testing. One such survey performed at 35 community clinics in the United States found the mean cost per rapid test was US$36.68 for HIV-negative clients and US$44.22 for preliminary-positive clients[30]. The difference in costs between Zambia and the US is likely related to increased operational and human resource costs in the US.

The high sensitivity and specificity of oral fluid based HIV testing along with its ease of use make it ideal to use in developing countries. The biggest challenge of integrating OraQuick® into testing algorithms is its cost. Ideally the increased cost of the OraQuick® test would be offset by an increase in the uptake of testing. However, our cost analysis was not able to model how many tests could be done and how many more HIV diagnoses could be made with OraQuick® testing because we lacked acceptability data regarding the OraQuick® tests. Moreover, OraQuick®’s acceptability versus blood-based rapid HIV testing in the literature is scant. There is one cross sectional study by Choko et al. in Malawi that showed very high acceptability and accuracy rates among participants using oral supervised self testing[13]. They reported that 91.9% of participants opted to self test after a brief demonstration and the accuracy was 99.2%. Given the many barriers to HIV testing and with self testing on the horizon, oral based HIV testing can offer an acceptable alternative to blood based testing.

In addition to lacking acceptability data, this study was also limited by partial verification bias; our gold standard for the diagnosis of HIV infection was two rapid tests, based upon how HIV is diagnosed in Zambia. For discordant results, patients did not have a tiebreaker test in the field, but instead were referred to the local clinic. We did not have access to ELISA or western blot (WB). Moreover, antibody based tests like OraQuick®, Determine®, and Uni-Gold^TM^ have the disadvantage of missing acute HIV infection, which is a significant limitation in a high prevalence population. Accuracy may have also been affected by difficulties reading the tests under field conditions that would be absent in a more controlled laboratory setting. Third, the HIV test results were not blinded. All HIV tests were performed and interpreted by same person, which could have introduced incorporation bias. Knowledge of the first test result may have influenced how the second test result was interpreted. Lastly, the test sensitivities used for the cost analysis were taken from published literature that had used the tests among high HIV prevalence populations; however, despite this fact the costing trends were apparent, Determine® and if positive, then Uni-Gold^TM^ was the least expensive of the testing algorithms.

The results from this study can still be generalized to other resource limited settings where HIV screening has been rolled out. Although test performance may decrease under field conditions, our study shows that those decreases are subtle and are offset by the high seroprevalence of HIV in this setting, thereby increasing the positive predictive value of the OraQuick® test in places like Zambia. Moreover, a recently published meta-analysis by Pai et al.[27] shows that the positive predictive value of OraQuick® test in high prevalence populations (>1%) was 98.65% versus 88.55% in low prevalence populations (<1%).

The need for increased HIV testing is apparent, but the challenges of universal testing carry with it the uncertainty of universal treatment. While access to antiretroviral therapy has revolutionized the face of HIV/AIDS in many developing countries like Zambia, the ultimate question of when to treat is still debatable. As more studies are needed to answer this question, this study adds to the growing body of literature that shows that oral-based HIV rapid testing in Africa can perform well.

## Conclusions

An alternative HIV testing algorithm could include OraQuick® ADVANCE Rapid HIV-1/2 Antibody test which had a high sensitivity and specificity. The current Determine®/Uni-Gold^TM^ testing algorithm is the least expensive when compared to Determine®/OraQuick®, OraQuick®/Determine® and OraQuick®/Uni-Gold^TM^ in the Zambian setting. From our field experience, oral fluid based testing offers many advantages over blood-based testing, especially with self testing on the horizon.

## Abbreviations

ELISA, Enzyme linked immunosorbent assay; PDA, Personal digital assistant; VCT, Voluntary counseling and testing; WB, Western blot; ZAMSTAR, Zambia South Africa TB and AIDS reduction study.

## Competing interests

The opinions and conclusions expressed in this paper are those of the authors and do not reflect those of the funding agencies and participating institutions. The tests were supplied free of charge by OraSure Technologies, Inc., but they had no part in the writing of the manuscript. The authors declare that they have no competing interests.

## Authors’ contributions

DZ participated in statistical analysis and manuscript writing. LM participated in cost analysis and manuscript writing. MM participated in study design, coordination, and manuscript writing. KS participated in study design, coordination, and manuscript writing. AS participated in statistical analysis. VB participated in study design, coordination, and manuscript writing. BK participated in conception, acquisition of test kits, coordination, supervision of rapid testing, and manuscript writing. PD participated in coordination, supervision of rapid testing, and manuscript writing. HA participated in conception, study design, coordination, and manuscript writing.

## Pre-publication history

The pre-publication history for this paper can be accessed here:

http://www.biomedcentral.com/1471-2334/12/183/prepub

## References

[B1] Ministry of Health Adult and adolescent antiretroviral therapy protocols 2010 Lusaka: Government of the Republic of Zambia

[B2] Joint United Nations Program on HIV/AIDS (UNAIDS)- World Health Organization(WHO) Revised recommendations for the selection and use of HIV antibody tests Wkly Epidemiol Rec 1997 72 81 87 9238418

[B3] McKenna SL Muyinda GK Roth D Mwali M Ng'andu N Myrick A Luo C Priddy FH Hall VM von Lieven AA Sabatino JR Mark K Rapid HIV testing and counseling for voluntary testing centers in Africa AIDS 1997 11 Suppl. 1 S103 S110 9376093

[B4] Global report: UNAIDS report on the global AIDS epidemic 2010 http://www.unaids.org/documents/20101123_globalreport_em.pdf

[B5] Shisana O, Rehle T, Simbayi LC, Zuma K, Jooste S, Pillay-van-Wyk V, Mbelle N, Zyl J, Parker W, Zungu NP, Pezi S South African national HIV prevalence, incidence, behaviour and communication survey 2008: a turning tide among teenagers? 2009 Cape Town: HSRC Press

[B6] Shisana O Rehle T Simbayi L Parker W Zuma K Bhana A Connolly C Jooste S Pillay V South African national HIV prevalence, HIV incidence, behaviour and communication survey, 2005 2005 Cape Town: HSRC Press

[B7] Shisana O Simbayi L Nelson Mandela/HSRC study of HIV/AIDS: South African national HIV prevalence, behavioural risks and mass media household survey 2002 2005 Cape Town: HSRC Press

[B8] Ministry of Health Kenya AIDS indicator survey 2007: preliminary report 2008 Nairobi: Government of Kenya

[B9] WHO: Toward Universal Access: Scaling Up Priority HIV/AIDS interventions in the health sector 2009 Geneva http://www.cdc.gov/hiv/topics/testing/rapid/pdf/RT_Purchasing-Chart_2-4-08.pdf

[B10] Morin SF Khumalo-Sakutukwa G Charlebois ED Removing barriers to knowing HIV status. Same-day mobile HIV testing in Zimbabwe J Acquir Immune Defic Syndr 2006 41 2 218 224 10.1097/01.qai.0000179455.01068.ab 16394855

[B11] Scully C Porter S HIV topic update: oro-genital transmission of HIV Oral Dis 2000 6 2 92 98 10702785 10.1111/j.1601-0825.2000.tb00107.x

[B12] Campo J Perea MA del Romero J Cano J Hernando V Bascones A Oral transmission of HIV, reality or fiction? an update Oral Dis 2006 12 3 219 228 10.1111/j.1601-0825.2005.01187.x 16700731

[B13] Choko AT Desmond N Webb EL Chavula K Napierala-Mavedzenge S Gaydos CA Makombe SD Chunda T Squire SB French N The uptake and accuracy of oral kits for HIV self-testing in high HIV prevalence setting: a cross-sectional feasibility study in Blantyre, Malawi PLoS Med 2011 8 10 e1001102 10.1371/journal.pmed.1001102 21990966 PMC3186813

[B14] Center for Disease Control: FDA-Approved Rapid HIV Antibody Screening Tests-Purchasing Details 2011 Atlanta http://www.cdc.gov/hiv/topics/testing/rapid/pdf/RT_Purchasing Chart_2-4-08.pdf

[B15] Pavie J Rachline A Loze B Niedbalski L Delaugerre C Laforgerie E Plantier JC Rozenbaum W Chevret S Molina JM Sensitivity of five rapid HIV tests on oral fluid or finger-stick whole blood: a real-time comparison in a healthcare setting PLoS One 2010 5 7 e11581 10.1371/journal.pone.0011581 20657834 PMC2906506

[B16] Kshatriya R Cachafeiro AA Kerr RJ Nelson JA Fiscus SA Comparison of two rapid human immunodeficiency virus (HIV) assays, Determine® HIV-1/2 and OraQuick® Advance Rapid HIV-1/2, for detection of recent HIV seroconversion J Clin Microbiol 2008 46 10 3482 3483 10.1128/JCM.00665-08 18685013 PMC2566129

[B17] Akanmu AS Akinsete I Adesemoye EF Okany CC Evaluation of saliva-based diagnostic test kit for routine detection of antibodies to HIV Afr J Med Med Sci 2001 30 4 305 308 14510108

[B18] Pascoe SJ Langhaug LF Mudzori J Burke E Hayes R Cowan FM Field evaluation of diagnostic accuracy of an oral fluid rapid test for HIV, tested at point-of-service sites in rural Zimbabwe AIDS Patient Care STDS 2009 23 7 571 576 10.1089/apc.2008.0225 19530953 PMC2856437

[B19] Piwowar-Manning EM Tustin NB Sikateyo P Kamwendo D Chipungu C Maharaj R Mushanyu J Richardson BA Hillier S Brooks Jackson J Validation of rapid HIV antibody tests in 5 African countries J Int Assoc Phys AIDS Care (Chic) 2010 9 3 170 172 10.1177/1545109710368151 PMC2989535 20530471

[B20] Eller LA Eller MA Ouma BJ Kataaha P Bagaya BS Olemukan RL Erima S Kawala L de Souza MS Kibuuka H Large-scale human immunodeficiency virus rapid test evaluation in a low-prevalence ugandan blood bank population J Clin Microbiol 2007 45 10 3281 3285 10.1128/JCM.00894-07 17699650 PMC2045340

[B21] Mao A Obi-Gwacham C Hill T Ryan C Hendricks A McKellar M Acute dengue fever causes false-positive reactivity in OraQuick® rapid HIV-1/2 antibody test J Acquir Immune Defic Syndr 2010 55 5 641 10.1097/QAI.0b013e3181f5b291 21931283 PMC4885708

[B22] Ayles HM Sismanidis C Beyers N Hayes RJ Godfrey-Faussett P ZAMSTAR, the Zambia South Africa TB and HIV reduction study: design of a 2 x 2 factorial community randomized trial Trials 2008 9 63 10.1186/1745-6215-9-63 18992133 PMC2585552

[B23] Determine® HIV-1/2 http://www.bp.co.th/events/pdf/Package%20Insert%20Determine®%20HIV%20eng.pdf, Accessed February 15, 2012

[B24] OraQuick® ADVANCE HIV-1/2 rapid antibody test http://orasure.com/docs/pdfs/products/OraQuick®_advance/OraQuick®-Advance-Package-Insert-English.pdf, Accessed February 15, 2012

[B25] Uni-gold recombigen HIV http://www.fda.gov/downloads/BiologicsBloodVaccines/BloodBloodProducts/ApprovedProducts/PremarketApprovalsPMAs/ucm093428.pdf, Acccessed February 15, 2012

[B26] Drummond MF Schulper JM Torrance GW O'brian JB Stoddart LG Methods for the economic evaluation of health care programmes 2005 Oxford: Oxford University Press

[B27] Pai NP Balram B Shivkumar S Martinez-Cajas JL Claessens C Lambert G Peeling RW Joseph L Head-to-head comparison of accuracy of a rapid point-of-care HIV test with oral versus whole-blood specimens: a systematic review and meta-analysis Lancet Infect Dis 2012 12 5 373 380 10.1016/S1473-3099(11)70368-1 22277215

[B28] Tegbaru B Messele T Wolday D Meles PH Tesema D Birhanu H Tesfaye G Bond KB Martin R Rayfield MA Evaluation of rapid HIV test kits on whole blood and development of rapid testing algorithm for voluntary testing and counseling centers in Ethiopia Ethiop Med J 2004 42 4 267 276 16122118

[B29] Lyamuya EF Aboud S Urassa WK Sufi J Mbwana J Ndugulile F Massambu C Evaluation of simple rapid HIV assays and development of national rapid HIV test algorithms in Dar es Salaam, Tanzania BMC Infect Dis 2009 9 19 10.1186/1471-2334-9-19 19226452 PMC2650699

[B30] Pinkerton SD Bogart LM Howerton D Snyder S Becker K Asch SM Cost of OraQuick® oral fluid rapid HIV testing at 35 community clinics and community-based organizations in the USA AIDS Care 2009 21 9 1157 1162 10.1080/09540120902729940 20024775

